# Supplementary material for: Polyketide synthase gene clusters in the ericoid mycorrhizal fungus Oidiodendron maius: genome-wide prediction, expression profiling, and functional characterization of knockout mutants
Source: Front Plant Sci. 2026 Sep 16;17:1924430. doi: 10.3389/fpls.2026.1924430 (PMC13623903; doi:10.3389/fpls.2026.1924430)
Supplement: Supplementary file 1 [file SupplementaryFile1.zip › Supplementary Materials/Supplementary Folder 1.zip!/Oidma1_AssemblyScaffolds_Repeatmasked/knownclusterblast/cluster34/jgi.p_Oidma1_44383_mibig_hits.html]

| MIBiG Protein | Description | MIBiG Cluster | MiBiG Product | % ID | % Coverage | BLAST Score | E-value |
| --- | --- | --- | --- | --- | --- | --- | --- |
| AGN71623.1 | salicylate\_hydroxylase | BGC0000027 | Azaphilone\_biosynthetic\_gene\_cluster | 37 | 98.8262910798 | 262 | 1.1e-69 |
| EHA28235.1 | hypothetical\_protein | BGC0001143 | Azanigerone\_biosynthetic\_gene\_cluster | 35 | 99.765258216 | 224 | 2e-58 |
| CAP95403.1 |  | BGC0001404 | Sorbicillin\_biosynthetic\_gene\_cluster | 32 | 99.5305164319 | 218 | 1.9e-56 |
| CAB38889.1 | putative\_salicylate\_hydroxylase\_(putative\_secreted\_protein) | BGC0000315 | Calcium-dependent\_antibiotic\_biosynthetic\_gene\_cluster | 30 | 92.9577464789 | 160 | 3.6e-39 |
| ALA99209.1 | CreL | BGC0001295 | Cremeomycin\_biosynthetic\_gene\_cluster | 28 | 83.3333333333 | 142 | 1.3e-33 |
| AEE65479.1 | oxidoreductase | BGC0000223 | Fluostatin\_biosynthetic\_gene\_cluster | 28 | 84.7417840376 | 107 | 3.6e-23 |
| AIT42124.1 | putative\_monooxygenase | BGC0001221 | Marinophenazines\_biosynthetic\_gene\_cluster | 26 | 87.558685446 | 86 | 6.6e-17 |
